# Tips and tricks for successfully culturing and adapting human induced pluripotent stem cells

**DOI:** 10.1016/j.omtm.2021.10.013

**Published:** 2021-11-03

**Authors:** Rocío Castro-Viñuelas, Clara Sanjurjo-Rodríguez, María Piñeiro-Ramil, Silvia Rodríguez-Fernández, Isidoro López-Baltar, Isaac Fuentes-Boquete, Francisco J. Blanco, Silvia Díaz-Prado

**Affiliations:** 1 Cell Therapy and Regenerative Medicine Group, Department of Physiotherapy, Medicine and Biomedical Sciences, Faculty of Health Sciences, University of A Coruña (UDC), 15006 A Coruña, Galicia, Spain; 2 Institute of Biomedical Research of A Coruña (INIBIC), University Hospital Complex A Coruña (CHUAC), Galician Health Service (SERGAS), 15006 A Coruña, Galicia, Spain; 3 Human Movement Biomechanics Group (HMBG), Tissue Homeostasis and Disease (THD) Lab, Skeletal Biology and Engineering Research Center (SBE), KU Leuven, 3000 Leuven, Belgium; 4 Centro de Investigaciones Científicas Avanzadas (CICA), Agrupación estratégica CICA-INIBIC, University of A Coruña, 15008 A Coruña, Galicia, Spain; 5 Centro de Investigación Biomédica en Red (CIBER) de Bioingeniería, Biomateriales y Nanomedicina (CIBER-BBN), 28029 Madrid, Spain; 6 Laboratorio de Genética Molecular y Radiobiología, Centro Oncológico de Galicia, 15009 A Coruña, Spain; 7 Tissular Bioengineering and Cell Therapy Unit (GBTTC-CHUAC), Rheumatology Group, 15006 A Coruña, Galicia, Spain

**Keywords:** iPSCs, feeders, feeder-free, culture, troubleshooting, tips and tricks, protocols, cryopreservation, irradiation

## Abstract

Reprogramming somatic cells toward pluripotency became possible over a decade ago. Since then, induced pluripotent stem cells (iPSCs) have served as a versatile and powerful tool not only for basic research but also with the long-term goal of using them in human cell transplantation after differentiation. Nonetheless, downstream applications are frequently blurred by the difficulties that researchers have to face when working with iPSCs, such as trouble with clonal selection, *in vitro* culture and cryopreservation, adaptation to feeder-free conditions, or expansion of the cells. Therefore, in this article we aim to provide other researchers with practical and detailed information to successfully culture and adapt iPSCs. Specifically, we (1) describe the most common problems when *in-vitro* culturing iPSCs onto feeder cells as well as its possible troubleshooting, and (2) compare different matrices and culture media for adapting the iPSCs to feeder-free conditions. We believe that the troubleshooting and recommendations provided in this article can be of use to other researchers working with iPSCs and who may be experiencing similar issues, hopefully enhancing the appeal of this promising cell source to be used for biomedical investigations, such as tissue engineering or regenerative medicine applications.

## Graphical abstract

**Figure:**
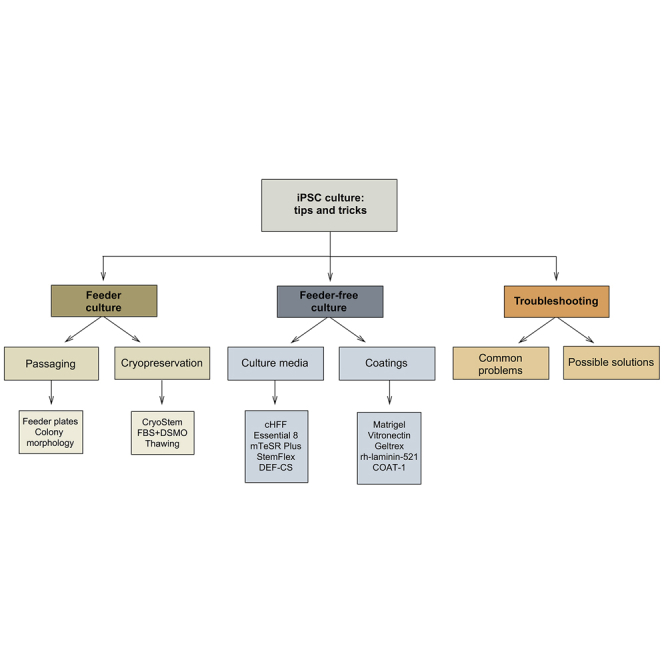

## Teaser

This article contains a descriptive methodological guide on how to culture iPSCs under feeder or feeder-free conditions, paying special attention to provide readers with detailed protocols, tips and tricks, as well as troubleshooting guides than can be of use to other researchers working with iPSCs.

## Background

Induced pluripotent stem cells (iPSCs) have the ability to proliferate indefinitely in culture without reduction of the quality, besides potential of differentiation into any desired cell type.^1^ Therefore, when first discovered in 2006,^2^ these cells were thought to be the “panacea” for biomedical applications. In the case of tissue engineering, iPSCs hold great potential for personalized tissues, which can be used for regenerative medicine and/or *in vitro* studies to tailor other medical interventions.^3^ However, all these advantages and potential applications are blurred by the difficulties that researchers have to face when working with iPSCs, such as trouble with clonal selection, *in vitro* culture, adaptation, and/or expansion of the cells.^4^ iPSCs' recovery after freezing is still an issue^5^^,^^6^ and, in addition, it has been reported that iPSC colonies may disappear and break up into single cells during initial colony morphology-based selection.^7^

The use of iPSCs in all downstream applications requires the establishment of protocols that will allow large-scale, cost-effective cultivation of cells, without compromising on their quality.^8^ Reprogramming methods and iPSC culture strategies initially involved the use of mouse or human feeder layers, thus coinciding with the protocol established by Thomson for *in vitro* culturing embryonic stem cells (ESCs).^9^ These feeder cells secrete essential growth factors, extracellular matrix components, and cytokines into the culture media, which support pluripotent cell growth and proliferation.^8^^,^^10^^,^^11^ Although a robust method, feeder-based systems are labor intensive, hard to scale,^11^ and can also be a source of animal pathogens and mycoplasma contamination.^8^^,^^11^^,^^12^ Moreover, this system does not allow the performance of all the required tests to characterize new-generated iPSC lines, since feeder cells can interfere with molecular and/or flow cytometry experiments.^13^ This is why in certain occasions iPSCs have to be “moved” to feeder-free culture systems. But when pluripotent cells are switched from one cell culture condition to another, they need to adapt to the new system to regain homeostasis^14^; this adaptation process is usually harmful for the cells, especially in the case of newly derived cell lines, often experiencing more differentiation and apoptosis than normal.^14^, ^15^, ^16^ Despite feeder-free reprogramming being proven feasible,^6^^,^^17^, ^18^, ^19^ feeder-based reprogramming methods are still the most common surface on which to develop the reprogramming process.^6^^,^^20^

In our group we have recently published the successful generation of three iPSC lines derived from human skin fibroblasts.^21^^,^^22^ It is worth mentioning that the problems that we had to face during the process of generation and characterization of the cells were numerous, especially regarding the adaptation to feeder-free conditions. There are currently a lot of published combinations of matrices and culture media to replace feeder layers (reviewed in Dakhore et al.,^8^ Sams and Powers,^8^, ^11^, ^13^, ^20^, ^23^, ^24^, ^25^ Skottman and Hovatta,^8^, ^11^, ^13^, ^20^, ^23^, ^24^, ^25^ Liu et al.,^8^, ^11^, ^13^, ^20^, ^23^, ^24^, ^25^ Anderson et al.,^8^, ^11^, ^13^, ^20^, ^23^, ^24^, ^25^ Nakamura et al.,^8^, ^11^, ^13^, ^20^, ^23^, ^24^, ^25^ Kishimoto et al.^8^, ^11^, ^13^, ^20^, ^23^, ^24^, ^25^). But when consulting the bibliography for troubleshooting, we could just find depthless explained methodology. It is widely known that generation and *in vitro* culture of iPSCs is a slow and tricky process,^8^^,^^26^ but the literature rarely explains the difficulties and problems found during the process of establishing new iPSC lines and daily working with them. Therefore, in this methodological article we aim to fill in this gap of knowledge by deeply describing the protocols we established for the complex process of generating iPSC lines. Special attention is paid to (1) which are the most common problems when *in-vitro*-culturing iPSCs onto feeder cells as well as its possible troubleshooting, and (2) comparing different matrices and culture media for adapting the iPSCs to feeder-free conditions.

We strongly believe that reporting this kind of problems and suggesting possible ways to have them solved will contribute to help other researchers that might have similar issues when working with iPSCs.

## Required materials

### Reagent list

• Basic fibroblast growth factor (bFGF) (13256029, Gibco Thermo Fisher Scientific)
• Bovine serum albumin (BSA) 9048-46-8, Sigma)
• Dimethyl sulfoxide (DMSO) (67-68-5, Sigma-Aldrich Química SA)
• Dulbecco's modified Eagle's medium (DMEM) (12-604F, Lonza)
• DMEM knockout without L-glutamine (KO-DMEM); (10829018, Gibco Thermo Fisher Scientific)
• DPBS with calcium and magnesium (14040133, Gibco Thermo Fisher Scientific)
• Fluorescence-activated cell sorting buffer (Becton Dickinson)
• Fetal bovine serum (FBS) (10082147, Gibco Thermo Fisher Scientific)
• Gentle cell dissociation reagent (07174, STEMCELL Technologies)
• Geltrex hESC-qualified, ready-to-use, reduced growth factor basement membrane matrix (A1569601, Gibco)
• GlutaMAX 100X (35050061, Gibco Thermo Fisher Scientific)
• Human foreskin fibroblasts, HFF-1 cell line (CRL2429, ATCC)
• Iscove’s modified Dulbecco medium (IMDM) (12440053; Gibco Thermo Fisher Scientific)
• Knockout serum replacement (10828028, Gibco Thermo Fisher Scientific)
• 2-Mercaptoethanol (50 mM; 31350010, Gibco Thermo Fisher Scientific)
• Matrigel basement membrane matrix (354234, Corning)
• Non-essential amino acids 100X (11140050, Gibco Thermo Fisher Scientific)
• Phosphate-buffered saline tablets (BR0014G, Oxoid)
• Phosphate-buffered saline with Ca^2+^ and Mg (14287, Thermo Fisher Scientific)
• Penicillin/streptomycin (P/S) (15140122, Gibco Thermo Fisher Scientific)
• Propidium iodide (PI) (P4170, Sigma-Aldrich)
• rh-Laminin-521 521 (A29249; Thermo Fisher Scientific)
• RNAse (500 μg/mL; Roche)
• RevitaCell 100X (A2644501; Gibco)
• ROCK inhibitor Y-27632 (04-0012, Stemgent)
• Saline serum (154 mmol/L Na and 154 mmol/L Cl^–^, Fresenius Kabi)
• StemFlex basal medium (A3349301, Gibco Thermo Fisher Scientific)
• StemFlex Supplement 10X (A3349201, Gibco Thermo Fisher Scientific)
• Trypan blue staining (T8154-20 ML, Sigma)
• TrypLE Select Enzyme (1X), no phenol red (12563011, Gibco Thermo Fisher Scientific)
• Versene solution (15040066, Gibco)
• 0.05% trypsin-EDTA (25300054, Gibco)
• 0.25% trypsin-EDTA (25200056, Gibco)

### Equipment list

• 0.22-μm filter (GSWP14250, Millipore)
• 15- and 50-mL conical tubes
• 175 μm “stripper” tips (Gynétics)
• Cryovials, 1.5 mL
• FACScalibur flow cytometer (Becton Dickinson) and CellQuest software (Becton Dickinson)
• CoolCell freezing container
• Neubauer chamber
• Polypropylene conical tubes (Corning Science)
• Real-time DS-FI2 photomicrograph camera
• Sight DS-L3 digital control monitor
• SMZ-745T stereomicroscope
• Stripper micropipette (Origio Midatlantic Devices)
• 100- and 150-mm tissue culture dishes
• Six-well tissue culture plates

### Reagent setup

• 5% DMEM medium: DMEM supplemented with 5% FBS and 1% P/S. Store at 4°C.
• bFGF: 100 μg/mL stock solution of bFGF in 2% filter sterilized BSA. Divide into usage size aliquots (50 μL). Store at −20°C.
• CRYOSTEM hPSC freezing medium (Cellseco Biological Industries).
• Freezing medium: 90% FBS and 10% DMSO. The use of freshly made freezing medium is recommended. Alternatively, freezing medium can be prepared and stored at 4°C in darkness up to 1 month.
• hES medium: KO-DMEM containing 20% knockout serum replacement, 1% non-essential amino acids, 1% GlutaMAX 100X, 1% P/S, 0.1 mM β-mercaptoethanol (0.5 mL of a 50 mM stock solution), and 100 μg/mL bFGF (all from Gibco). Store at 4°C and use within 1 month. Do not pre-warm the whole bottle.
• HFF medium: IMDM supplemented with 10% FBS and 1% P/S. Store at 4°C.
• Matrigel solution: thaw Matrigel basement membrane matrix slowly at 4°C overnight on ice. While working on ice inside a laminar flow hood, Matrigel solution is prepared at 1:40 dilution in P/S and KO-DMEM. Cool the pipette by aspirating cold KO-DMEM up and down 10–20 times. Using a cold pipette, aseptically transfer Matrigel to KO-DMEM. Invert the solution several times to obtain homogeneous Matrigel solution. Do not shake again and store at 4°C.
• rh-Laminin-521: thaw rh-laminin-521 between 2°C and 8°C, mix gently by inversion 3–5 times, divide into usage size aliquots (300 μL recommended) in polypropylene tubes and store at −20°C until expiration date. Avoid extended exposure of protein to ambient temperatures.
• ROCK inhibitor Y-27632: reconstitute in DMSO to prepare a stock solution of 10 mM and divide into aliquots of 10 μL. Store aliquots at −20°C up to 6 months.
• StemFlex complete medium: thaw the frozen StemFlex Supplement 10X at room temperature (RT) for ∼2 h or overnight at 4°C. Mix the thawed supplement by gently inverting 3–5 times. Aseptically transfer 50 mL of StemFlex Supplement 10X to the bottle of StemFlex basal medium. Gently invert the bottle several times to obtain an homogeneous complete medium. Aliquot the medium and store at −20°C for up to 6 months. To avoid multiple freeze-thaw cycles, we recommend preparing 25- and 50-mL aliquots and use as needed. Before use, warm complete medium required for that day at RT until it is no longer cool to the touch.
• Vitronectin (VTN-N) recombinant human protein, truncated (0.5 mg/mL). Thaw the vial of vitronectin at RT and prepare 60-μL aliquots of vitronectin in polypropylene tubes. Freeze the aliquots at −80°C or use immediately.

### Equipment setup

**Figure 1:**
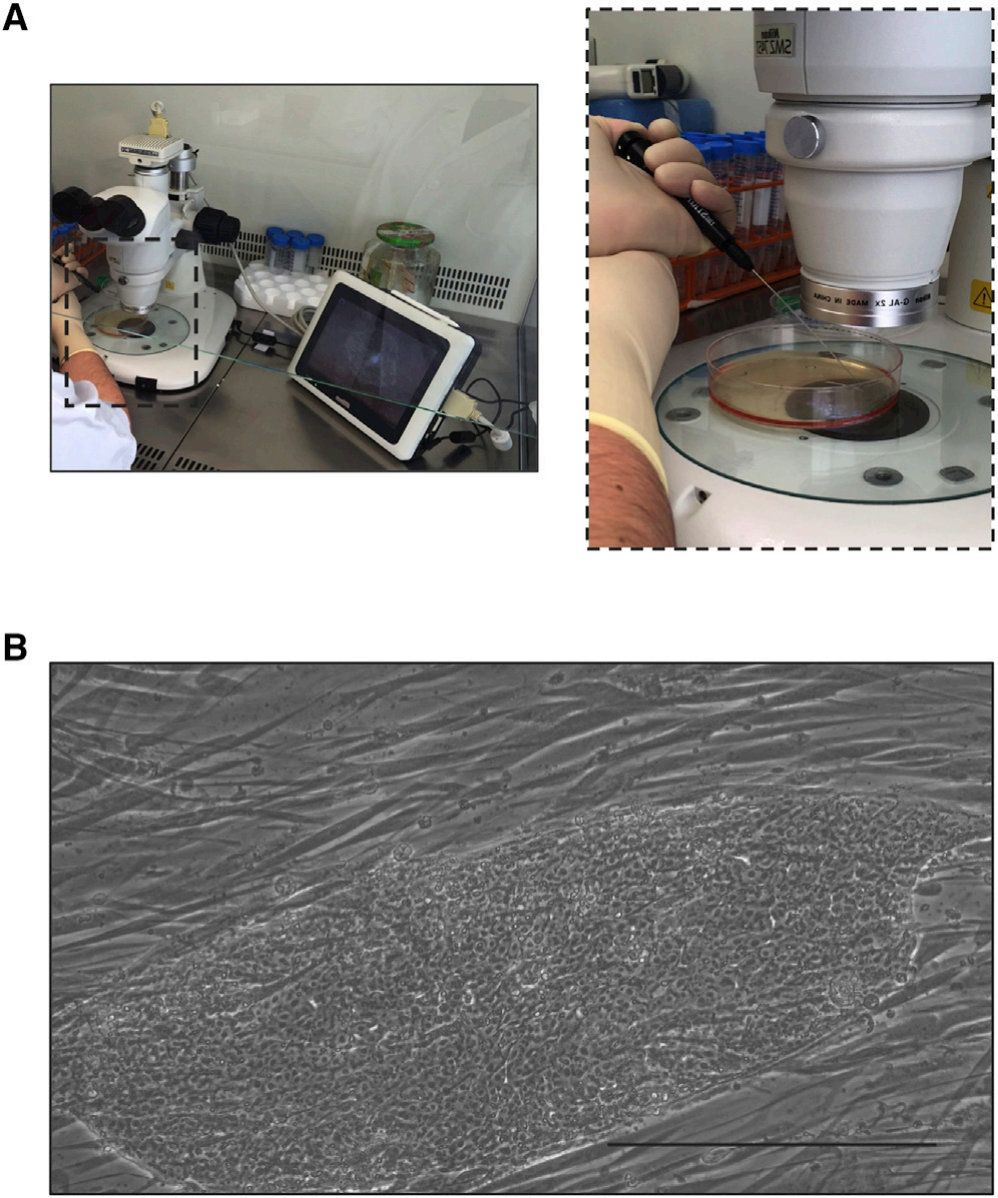
Picking iPSC colonies (A) Setup of the SMZ-745T stereomicroscope coupled to a real-time DS-FI2 photomicrograph camera and a Sight DS-L3 digital control monitor inside the laminar flow hood. (B) Representative image of one iPSC colony with the desired morphology cultured onto feeder cells. Scale bar, 50 μm.

• For the manual picking of iPSC colonies, equip a laminar flow hood with a stereomicroscope coupled to a real-time camera that allows for visualizing the iPSC colonies while working inside the laminar flow hood (Figure 1A).

## Experimental procedure

Note: all of these protocols must be performed in a sterile manner. All reagents, except for BSA, are suitable for cell culture and there is no need of filter sterilization.

*Note: optimization and testing of the protocols included in this manuscript has been performed using three iPSC lines previously generated in our laboratory: N1-FiPS4F#7, MOA1-FiPS4F#7, and MOA2-FiPS4F#17.* ^19^ ^,^ ^22^

### Preparation of a stock of feeder cells

*Note: generating a stock of feeder cells is necessary for performing cell reprogramming and sub-culturing of iPSC colonies. The protocol we recommend here is based on mitotically inactivating the foreskin fibroblast cell line HFF-1 with γ-irradiation, but other methods for arresting cell cycle such as mitomycin C can be used.* ^27^

#### Expansion of the cells and irradiation

1. Before performing the irradiation process, culture the HFF-1 cell line in IMDM supplemented with 10% FBS and 1% P/S (HFF medium), at 37°C in a humidified atmosphere with 5% CO_2_.
2. When cell confluence reaches 90%–100%, perform subcultures for cell expansion until sufficient numbers of cells (approximately 6–10, 150-cm^2^ culture dishes at 100% confluence) are obtained.

Note: do not over expand the HFF-1 before irradiation. Too many cells will result in too many frozen cryovials. We do not recommend using feeder cells that have been stored for longer than 3 months in liquid nitrogen since they tend to deteriorate. This makes the iPSC colonies more likely to break up into single cells and disappear in culture.

3. On the day of irradiation, remove the HFF medium from the plates and wash the cells twice with PBS.
4. Incubate the HFF-1 cell line with 0.25% trypsin-EDTA for 2–3 min at 37°C.
5. To verify that cells are not still adhered to the culture dish, visualize the plates through an inverted microscope.
6. When HFF-1 cells are detached from the culture dishes, inactivate the trypsin-EDTA with a double volume of 5% DMEM.
7. Collect cell suspension in a 50-mL conical tube and centrifuge for 7 min at 400 × *g* to obtain a cell pellet.
8. Resuspend the cell pellet in 50 mL of warm HFF medium.
9. Immediately irradiate the HFF-1-containing tube at 75 Gy. In this protocol we used a Varian Unique linear accelerator (Centro Oncológico de Galicia, Spain).

Note: if performing this protocol for the first time, and due to possible differences in the accelerators used, we recommend verifying that 75 Gy irradiation inhibits HFF-1 mitosis by flow cytometry as described below.

#### Cell-cycle evaluation by flow cytometry

Here, the cell cycle is evaluated by flow cytometry and using PI.

1. Transfer 2 × 10^5^ cells to polypropylene conical tubes and centrifuge at 400 × *g* for 5 min.
2. Wash the pelleted cells twice with saline serum.
3. Discard the supernatant and resuspend pelleted cells in a solution composed of 350 μL PI (stock concentration 1.0 mg/mL) and 1.5 μL RNAse (stock concentration 500 μg/mL) per tube.
4. Incubate the tubes in the dark for 15 min and then centrifuge for 5 min at 400 × *g*.
5. Resuspend the pellets in fluorescence-activated cell sorting buffer to a final volume of 200 μL.
6. Analyze the cells in a FACScalibur flow cytometer using the CellQuest software.

Note: non-irradiated HFF-1 cells must be also analyzed as negative control.

#### Cryopreservation of feeder cells

1. Count the irradiated HFF-1 cells using trypan blue staining and a Neubauer chamber.
2. Centrifuge the cell suspension for 7 min at 400 × *g*.
3. While centrifuging, prepare the necessary amount of freezing medium according to cell count and considering 1 mL for every 2 × 10^6^ cells, and place it on ice.
4. Resuspend pelleted cells in cold (2°C–8°C) freezing medium.

Note: we recommend adjusting the volume of freezing medium according to cell count to cryopreserve 2 × 10^6^ cell/mL.

*Note: at this point it is recommended to work as fast as possible (± 5 min) until the cryovials are introduced in the freezer since exposure of the cells to DMSO at RT is toxic for the cells.* ^28^

5. Introduce cryovials containing 1 mL of the cell suspension in a freezing container (e.g*.*, CoolCell) and store at −80°C for 24 h.
6. After 24 h, transfer the cryovials to liquid nitrogen until used.

#### Thawing of feeder cells

1. Thaw feeder cells by quickly introducing the cryovials in a 37°C water bath.

*Note*: *the water bath should be cleaned periodically with addition of water bath disinfectants (e.g., Sigmaclean).*

2. Gently shake the cryovial until only a small piece of ice remains.

Note: do not completely submerge the cryovial in the water bath or contamination of the feeders may occur.

3. At this point, spray the cryovial with 70% ethanol and place inside the laminar flow hood.
4. Collect the cell suspension with a micropipette and transfer in a dropwise manner to a 15-mL conical tube containing pre-warmed 5% DMEM.
5. To remove the freezing medium, centrifuge tubes with the cell suspension at 400 × *g* for 5 min.
6. Discard the supernatant and resuspend pelleted cells in 2 mL/well of hESC medium to seed the feeders in six-well culture dishes (see “Culture of iPSCs onto feeder cells”).

Note: we recommend that one cryovial with 2 × 10^6^ cells be used to prepare four to five confluent wells of a six-well culture dish (cell density of ±4 × 10^4^ cells/cm^2^).

### Culture of iPSCs onto feeder cells

#### Seeding of feeder cells on six-well plates

1. For preparation of the six-well plates, add 1.5 mL of cold Matrigel solution (see “reagent setup,” for tips on how to prepare Matrigel solution) to each well, ensuring that the solution completely covers the bottom of the dish, and incubate for at least 1 h at RT inside a laminar flow hood.
2. Approximately 10 min before the 1-h incubation finishes, thaw the feeder cells (see “Preparation of a stock of feeder cells,” thawing of feeder cells).
3. Aspirate Matrigel solution and seed the feeder cells as described above.

Note: remove Matrigel solution completely but paying attention not to damage the coating of the wells.

4. Move the six-well plates in a cross-shaped manner to ensure evenly distribution of the feeder cells.
5. Incubate the plates with feeder cells in 2 mL/well of hESC medium for 48 h at 37°C, normoxia, and 5% CO_2_ before seeding the iPSCs colony fragments to allow the feeder cells to settle in culture and avoid unwanted spreading over the iPSCs.

#### Picking iPSC colonies

1. Identify the iPSC colonies with the right morphology (i.e., compact colonies with well-defined edges and comprised of cells with high nucleus/cytoplasm ratio)^29^^,^^30^ and that grow robustly (Figure 1B).
2. Prepare and turn on the stereomicroscope.
3. Prepare the stripper micropipette and its 175-μm tips.
4. Replace hESC medium with 2 mL of fresh warm hESC medium in the six-well feeder plates that have been prepared 48 h in advance. The number of wells prepared should be adjusted depending on the number of ready to pick colonies considering a general 6:1 ratio, meaning six healthy iPSC colonies per new well of a six-well plate.

Note: when refreshing the hESC medium of the wells to which colony fragments will be transferred, leave ±0.5 mL of old medium in the well. This will help to keep the growth factors and cytokines secreted by the feeder cells.

Note: do not prepare the plates more than 48 h in advance.

5. Bring both six-well plates (i.e*.*, the one containing the iPSC colonies to be picked and the one containing the new feeder cells) to the laminar flow hood.
6. Carefully break up the selected iPSC colonies into small fragments while lifting and aspirating them by using a stripper micropipette and its 175-μm tips, as shown in Figure 1A and Video S1.

Note: to ensure healthy iPSCs cultures, it is of uttermost importance not to pick the differentiated areas of the colonies.

7. Gently transfer fragments of colonies into a new well with feeder cells in a 6:1 ratio, meaning six healthy iPSC colonies per new well of a six-well plate.

Note: this ratio allows iPSC colonies to grow within 5–9 days in culture and with enough space to avoid touching colonies and, as such, unwanted differentiation. Please, consider that empirical determination of the optimal ratio may be required.

8. Once a six-well plate is completed, transfer it to the incubator and delicately move it in a cross-shaped manner to homogeneously distribute the fragments.
9. Leave the plates undisturbed inside the incubator for 48 h.
10. From 48 h onward, replace hESC medium daily.

Note: we recommend not aspirating all the medium, but to leave ±0.5 mL of medium before adding 2 mL of fresh hESC medium.

11. Clonal iPSCs lines are established by manually picking human ESC-like colonies every 5–9 days, depending on the clone (Figure 1B).

Note: these time values differ in comparison with the growth period before the first split, after reprogramming of the somatic cells. In this case, iPSC colonies should be first picked after 26–32 days in culture, depending on the clone.

Video S1. Procedure to harvest colony fragments for passaging and expanding the iPSCs. It shows the procedure followed to harvest colony fragments for passaging and expanding the iPSCs. As described in the manuscript, iPSC-colonies are carefully broken up in small fragments, lifted and aspirated while working inside the laminar flow as shown.

### Creating the iPSC batch

#### Freezing iPSCs

Note: in this study, we tested two types of freezing media, one commercially available (CRYOSTEM, Stem Cell; Option A) and one prepared in our laboratory (freezing medium; see reagent setup section; Option B).

Note: for both option A and option B, the remaining colonies from previously picked wells are kept, and leftover colonies are picked again for freezing. Also, when the iPSC lines are established (approximately at passages 10–15) the split ratio can be doubled and wells with colonies that had not been previously picked can be used entirely for freezing.

1. Proceed as described in the section above (“Picking iPSC colonies”) until step number 3.
2. Bring the selected six-well plate to the laminar flow hood.
3. Carefully break up all the colonies (typically ±10) in each well as shown in Video S2.
4. Aspirate independently the hESC medium containing the colony fragments of each well with a 5-mL pipette and transfer the suspension to 15-mL conical tubes.
5. Centrifuge the suspensions at 280 × *g* for 2–4 min at RT.
6. While centrifuging, prepare and label the required number of cryovials (i.e., one cryovial per well to be frozen).
7. Carefully aspirate the supernatant paying attention to keep the cell pellet intact.
8. Gently tap the tubes with pelleted iPSCs, hanging the tube with one hand and tapping the bottom of the tube with the other, moving the fingers in a sequential manner to smoothly dislodge the cell pellet from the tube bottom.
9. Resuspend each iPSCs pellet in 0.5 mL of cold (4°C) CRYOSTEM/freezing medium and carefully pipette up and down four to five times with a 5-mL pipette until cell clumps are uniformly suspended.
10. Transfer the cell suspension to 1.5-mL cryovials with a 5-mL pipette.

*Note: at this point, the cells are in contact with DMSO, and they should therefore be quickly aliquoted and frozen (±5 min) since exposure of the cells to DMSO at RT is toxic for the cells.* ^28^

11. Introduce the cryovials containing the cell suspension in a freezing container and store at −80°C for 24 h.
12. Transfer the cryovials to a liquid nitrogen tank for long-term storage.

Note: we recommend performing this process (option A or option B) after every passage, until enough backup vials (approximately five vials per line) have been frozen.

Video S2. Procedure to harvest colony fragments for cryopreserving iPSCs. It shows the procedure followed to harvest colony fragments for cryopreserving. As described in the manuscript, the followed protocol was similar to the one described for picking iPSCs. However, instead of aspirating and transferring each small fragment of the colonies, all the colonies remaining in each well were carefully broken up and aspirated altogether.

#### Thawing iPSCs

1. Prepare 10 mL of pre-warmed (37°C) hESC medium containing 1 μL/mL of 10 mM Rho-associated protein kinase (ROCK) inhibitor Y-27632 (final concentration 10 μM) in 15-mL conical tubes.
2. Take the desired frozen vials of iPSCs from the liquid nitrogen tank and keep on ice until reaching the water bath.
3. Quickly thaw the iPSCs (<1 min) by gently swirling the vial in the 37°C water bath, until just a small bit of ice is left in the vial.
4. At this point, spray the cryovials with 70% ethanol and place inside the laminar flow hood.
5. Open the cryovials and add 1 mL of pre-warmed hESC medium in a dropwise manner.
6. Transfer the 1-mL cell suspension to the 15-mL conical tubes prepared in step 1.

Note: pipette the iPSCs carefully, so that cell clumps are not broken into single cells.

7. Centrifuge the tubes at 200 × *g* for 4 min.
8. Aspirate the supernatant and gently resuspend pelleted cell clumps in 1 mL of pre-warmed hESC medium.

Note: 1 mL of the iPSC suspension is transferred to one well of a six-well plate seeded with feeder cells and already containing 1 mL of hESC medium +1 μL/mL ROCK inhibitor (final concentration 10 μM).

9. Place the six-well culture dishes containing the thawed iPSCs in the incubator (37°C and 5% CO_2_) and leave undisturbed for 48 h.
10. From 48 h onward, replace the 2 mL of hESC medium every day.

Note: we recommend not aspirating all the medium but leaving ±0.5 mL of medium before adding 2 mL of fresh hESC medium

11. Check the culture plates daily under the microscope to identify iPSC colonies.

### Adaptation to feeder-free conditions

Note: in this methodological article, we describe and test different culture media (either commercial or “lab-made”) combined with different matrix coatings and subculture methods, to determine the best method to adapt the generated iPSCs to feeder-free conditions (Table 1).

**Table 1:** Summary of the different matrices and culture media tested for the adaptation of the iPSCs to feeder-free culture conditions

Matrix	Culture medium	Results
Matrigel	cHFF^a^	fragments of colonies adhered after the first manual passage but not after the second one
	Essential 8^b^	fragments of colonies adhered after the first manual passage but differentiated areas appeared
	mTeSR Plus^b^	good colony morphology; low cell death; possible to expand without differentiation
Vitronectin	Essential 8^b^	fragments of colonies did not adhere
	mTeSR Plus^b^	fragments of colonies adhered to the culture dishes, but debris and differentiated cells were observed
Geltrex	Essential 8^b^	fragments of colonies did not adhere
	StemFlex^b^	fragments of colonies did not adhere
DEF-CS	DEF-CS	a number of clones lost the ESC-like colony morphology
rh-Laminin-521	StemFlex^b^	after a number of passages, some clones lost the ESC-like colony morphology

a Tested with and without 1 μL/mL of ROCK inhibitor Y-27632.

b Tested with and without 10 μL/mL of RevitaCell 100X. cHFF, conditioned medium from non-dividing human foreskin fibroblasts; ESC, embryonic stem cell.

#### Lab-made culture media

Note: lab-made media is obtained from conditioned media, which is prepared by collecting supernatants from a monolayer of mitotically inactivated HFF (cHFF) as described below.

1. Follow the protocol described in “Preparation of a stock of feeder cells” (see also “Expansion of the cells and irradiation”) to mitotically inactivate HFF-1 cells.
2. Seed the irradiated HFF-1 cells in 100-mm culture dishes coated with Matrigel solution to have a confluency of 95%–100%, and culture them in hESC medium without bFGF.

Note: to prepare Matrigel-coated dishes see “Culture of iPSCs onto feeder cells” (see also “Seeding of feeder cells on six-well plates”). For recommended volumes in a 100-mm dish refer to Table 2.

3. Every 24 h, refresh the medium of the 100-mm plates keeping the conditioned medium in a sterile bottle and store it at 4°C. Conditioned medium without bFGF can be stored at 4°C for up to 2 months.
4. Before its usage, pass the conditioned medium through a 0.22-μm filter to remove cell debris and add 1 μg/mL of bFGF.

**Table 2:** Recommended volumes for the different reagents used

Reagent	Culture vessel	Approximate surface area	Recommended volume
Matrigel	6-well plate	10 cm^2^/well	2 mL
	12-well plate	4 cm^2^/well	0.8 mL
	24-well plate	2 cm^2^/well	0.4 mL
	35-mm dish	10 cm^2^	2 mL
	60-mm dish	20 cm^2^	4 mL
	100-mm dish	60 cm^2^	12 mL
Vitronectin	6-well plate	10 cm^2^/well	1 mL/well
	12-well plate	4 cm^2^/well	0.4 mL/well
	24-well plate	2 cm^2^/well	0.2 mL/well
	35-mm dish	10 cm^2^	1 mL
	60-mm dish	20 cm^2^	2 mL
	100-mm dish	60 cm^2^	6 mL
Rh-Laminin-521	6-well plate	10 cm^2^/well	2 mL
	12-well plate	4 cm^2^/well	0.8 mL
	24-well plate	2 cm^2^/well	0.4 mL
	35-mm dish	10 cm^2^	2 mL
	60-mm dish	20 cm^2^	4 mL
	100-mm dish	60 cm^2^	12 mL
Geltrex	6-well plate	10 cm^2^/well	1.5 mL
	12-well plate	4 cm^2^/well	0.6 mL
	24-well plate	2 cm^2^/well	0.3 mL
	35-mm dish	10 cm^2^	1.5 mL
	60-mm dish	20 cm^2^	3 mL
	100-mm dish	60 cm^2^	6 mL
COAT-1	6-well plate	10 cm^2^/well	1.5 mL
	12-well plate	4 cm^2^/well	0.6 mL
	24-well plate	2 cm^2^/well	0.3 mL
	35-mm dish	10 cm^2^	1.5 mL
	60-mm dish	20 cm^2^	3 mL
	100-mm dish	60 cm^2^	6 mL
Versene/gentle cell dissociation reagent/trypsin/TrypLE Select Enzyme	6-well plate	10 cm^2^/well	1 mL
	12-well plate	4 cm^2^/well	0.4–1 mL
	24-well plate	2 cm^2^/well	0.2–0.3 mL
	35-mm dish	10 cm^2^	1 mL
	60-mm dish	20 cm^2^	3 mL
	100-mm dish	60 cm^2^	5 mL
Culture media	6-well plate	10 cm^2^/well	2 mL
	12-well plate	4 cm^2^/well	1–2 mL
	24-well plate	2 cm^2^/well	0.5–1 mL
	35-mm dish	10 cm^2^	2 mL
	60-mm dish	20 cm^2^	5 mL
	100-mm dish	60 cm^2^	12 mL

#### Commercial culture media

Besides lab-made culture media, four different commercial media were tested in this study, following the manufacturer's instructions: Essential 8 (Thermo Fisher Scientific, Spain), StemFlex (Gibco, Thermo Fisher Scientific), mTeSR Plus (STEMCELL Technologies), and Cellartis defined culture system (DEF-CS) (Takara Bio).

#### Matrix coatings and culture media combinations

Vitronectin:

1. Thaw one vitronectin aliquot at RT.
2. Prepare vitronectin by diluting it in PBS without Ca^2+^ and Mg at RT in a 1:100 ratio as recommended by the manufacturer (i.e., 60 μL of vitronectin in 6 mL of PBS without Ca^2+^ and Mg). Gently resuspend by pipetting up and down.
3. Add 1 mL of vitronectin solution to each well of a six-well plate, ensuring that all the surface is completely covered.

Note: for recommended volumes in other culture surfaces refer to Table 2.

4. Incubate for 1 h at RT.
5. After the 1-h incubation, aspirate the vitronectin solution and discard. No rinsing is needed.
6. Immediately plate the iPSCs in 2 mL/well of pre-warmed cell culture medium.

Matrigel matrix:

1. Prepare the Matrigel solution as described in the “reagent setup” section.
2. Prepare Matrigel-coated dished as described in “Culture of iPSCs onto feeder cells”(see also “Seeding of feeder cells on six-well plates”). For recommended values in other culture surfaces refer to Table 2.
3. Add 2 mL/well of culture medium before seeding the iPSCs.

rh-Laminin-521:

Note: the optimal concentration of rh-laminin-521 should be determined empirically. Here, we use a working concentration of 2.5 μg/mL as recommended by the manufacturer and the instructions are given to coat a six-well plate. For recommended volumes in other culture surfaces refer to Table 2.

1. To coat a six-well plate, slowly thaw one aliquot (300 μL) of the −20°C rh-laminin-521 stock (see “reagent setup”) between 2°C and 8°C.
2. Once thawed, add the 300 μL of rh-laminin-521 to a 15-mL conical tube containing 12 mL of PBS with Ca^2+^ and Mg and gently resuspend by pipetting up and down.
3. Add 2 mL of the rh-laminin-521 solution to each well of a six-well plate, ensuring all the surface is completely covered.
4. Incubate at 37°C for at least 2 h.
5. Carefully aspirate the rh-laminin-521 solution with a micropipette and discard. No rinsing is needed.
6. Immediately plate the iPSCs in 2 mL/well of pre-warmed cell culture medium.

Geltrex:

1. Prepare Geltrex solution (1:100) in KO-DMEM with P/S (i.e., 60 μL of Geltrex in 6 mL of KO-DMEM with P/S). Gently resuspend by pipetting up and down.

Note: empirical determination of Geltrex concentration may be required.

2. Add 1 mL of Geltrex solution to each well of a six-well plate, ensuring that all the surface is completely covered.

Note: for recommended volumes in other culture surfaces refer to Table 2.

3. Incubate 1 h at 37°C followed by 1 h at RT.
4. Aspirate the Geltrex coating and immediately plate the iPSCs in 2 mL/well of pre-warmed cell culture medium. No rinsing is needed.

COAT-1:

Note: COAT-1 is the coating from the DEF-CS kit.

1. Prepare COAT-1 by diluting it in a 1:20 proportion in PBS with Ca^2+^ and Mg and mix gently and thoroughly by pipetting up and down.
2. Add 1.5 mL of COAT-1 solution to each well of a six-well plate, ensuring that all the surface is completely covered. For recommended volumes in other culture dishes refer to Table 2.
3. Incubate the dishes at 37°C overnight.
4. The following day, carefully remove the remaining solution and immediately plate the iPSCs in 2 mL/well of pre-warmed DEF-CS cell culture medium. No rinsing is needed.

Note: before adding DEF-CS culture medium, incorporate growth factors 1, 2, and/or 3 following the manufacturer's instructions.

#### Cell passaging for feeder-free adaptation of iPSCs

1. Prepare the culture dishes in advance as described in the previous section (“Matrix coatings and culture media combinations”).
2. Identify the iPSC colonies that are ready to pick and proceed as described in “Culture of iPSCs onto feeder cells” (see also “Picking iPSC colonies”) until step number 7.
3. Carefully transfer the iPSC fragments from 10 to 12 colonies to each well of a 6-well plate containing 2 mL/well of culture medium supplemented with 1μL/mL (10 μM) of 10 mM ROCK inhibitor Y-27632.

Note: as a start point, picking and seeding the double of colonies per well is recommended since cell death is normally high after the first passage to feeder-free culture conditions.

Note: when using the DEF-CS kit, no ROCK inhibitor was added to the culture medium.

4. Once a six-well plate is completed, bring it back to the incubator and delicately move it in a cross-shaped manner to homogeneously distribute the fragments.
5. Let the colonies grow with daily medium changes.

Note: it is possible to change the medium immediately after the first 24 h post seeding.

#### Cell passaging for feeder-free sub-culturing of iPSCs

1. Perform sub-culturing of the iPSCs once the colonies are grown and with a confluency of approximately 80%.
2. For the first two passages after feeder-free adaptation of the iPSCs, manually split the colonies using the stripper micropipette as described in “Culture of iPSCs onto feeder cells” (see also “Picking iPSC colonies”).
3. From the second passage onward, different reagents can be used to detach the cells, either in clamps (option A) or single cells (option B), depending on the experimental needs.
4. To obtain clamps of cells (option A) incubate the iPSC-containing plates with non-enzymatic Versene solution for 3–5 min or with gentle cell dissociation reagent for 8 min at 37°C. For recommended volumes refer to Table 2.
5. Carefully remove Versene or gentle cell dissociation reagent after the incubation time.

Note: remove Versene or gentle cell dissociation reagent carefully so the cells are not detached from the culture dish.

6. Gently detach the iPSC colonies by flushing fresh warm culture medium.

Note: when flushing fresh culture medium, we recommend doing it with a 5- to 10-mL pipette, so that the cell clumps are not broken into single cells.

7. Aspirate the cell clump suspension with a 5- to 10-mL pipette and transfer it to a 15-mL conical tube and gently pipette it up and down 5–7 times before transferring to a new plate to ensure that the size of the fragments is appropriate.

Note: colony fragments obtained with gentle cell dissociation reagent should be transferred directly to the new plate.

8. Let the cell clumps spontaneously sediment by gravity at the bottom of the tube (≤5 min) and remove supernatant without disturbing the pelleted clumps.
9. Resuspend the iPSC fragments in the appropriate amount of warm medium (i.e., 2 mL/well of a six-well plate) and seed in a bigger surface for cell expansion. For recommended volumes in other culture surfaces refer to Table 2.

Note: we recommend starting with a split ratio of 1:2 or 1:3, meaning the iPSC colonies are contained in one well to two or three new wells of a six-well plate, and subsequently increase this ratio as the feeder-free cultures of iPSCs are more established.

10. To obtain a single-cell suspension (option B), incubate the iPSCs with TrypLE Select Enzyme 1X diluted 1:10 in PBS, for 5 min at 37°C.
11. After the 5-min incubation, inactivate TrypLE Select Enzyme 1X by gently adding one volume of fresh warm culture medium.
12. Carefully collect the cell suspension in a 15-mL conical tube.

Note: cells can be counted using trypan blue and a Neubauer chamber.

13. Centrifuge the tube at 280 × *g* for 5 min to obtain a cell pellet and resuspend in the desired volume of medium for performing downstream analysis.

Note: better survival of single iPSCs has been observed when seeded in rh-laminin-521-coated dishes.

## Outcomes

### Generation of feeder cells and subsequent iPSC culture

**Figure 2:**
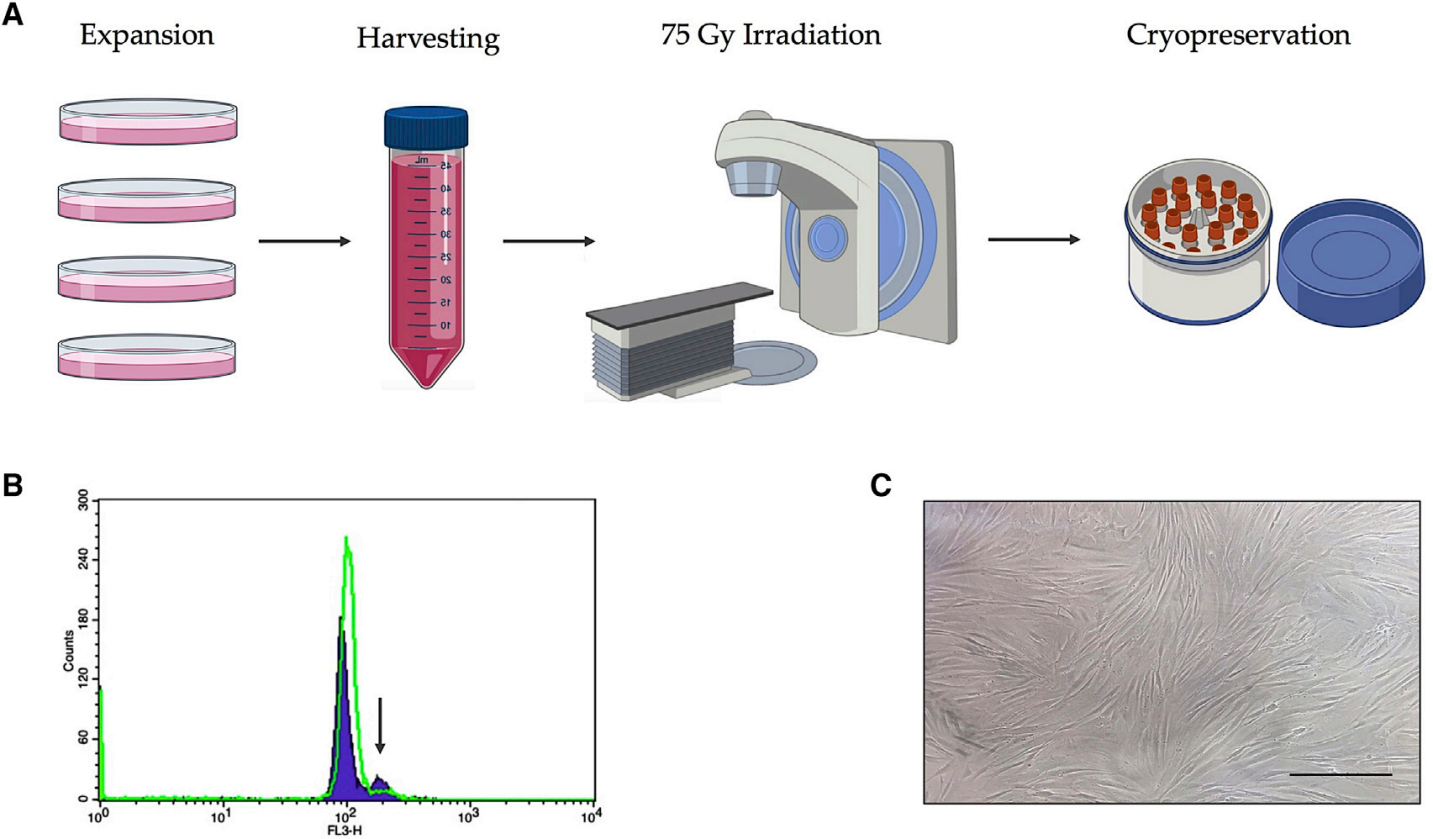
Generation of feeder cells (A) Scheme representing the followed workflow to generate the stock of feeder cells. Scheme prepared with BioRender. (B) Histogram representing the results of the cell-cycle analysis by flow cytometry. Irradiation of the foreskin fibroblast cell line HFF-1 at 75 Gy (green line) caused cell-cycle arrest (arrow), in comparison with unirradiated HFF-1 cells (purple area). (C) Homogeneous monolayer of feeder cells at >80% confluency. Scale bar, 200 μm.

Cell-cycle analysis showed that the irradiation of the foreskin fibroblast cell line HFF-1 at 75 Gy caused cell-cycle arrest of irradiated HFF-1 cells, in comparison with unirradiated HFF-1 cells (Figure 2B). Therefore, this radiation dose together with the protocol described above were established as effective to generate the stocks of feeder cells. The number of feeder cells that were seeded in the culture dishes for culturing and expanding the iPSC colonies was adjusted to achieve a homogeneous monolayer of feeder cells at >80% confluency (Figure 2C). To achieve this, a total of 2 × 10^6^ feeder cells were seeded in five wells of a six-well plate (cell density of ±4 × 10^4^ cells/cm^2^).

Regarding the iPSC culture onto feeder cells, it was observed that, using the protocol described in the 'culture of iPSCs onto feeder cells' section, fragments of iPSC colonies after picking were able to form new colonies onto feeder cells. These new colonies displayed the desired typical iPSC morphology (Figure 1B): compact colonies with well-defined edges and comprised of cells with high nucleus/cytoplasm ratio^29^^,^^30^ and were ready to pick every 5–9 days, depending on the clone.

#### Troubleshooting

Problem:

• iPSC colonies are not growing after passaging or they spontaneously differentiate.

Possible reasons/solution:

• Differentiated colonies or areas of colonies are common at early passages. Avoiding picking the differentiated colonies/areas and transferring only undifferentiated colonies to a new plate will increase the quality of the cultures. It is very likely that the number of colonies to be picked needs to be increased right after the reprogramming.
• The quality of feeders is absolutely crucial to maintain the iPSCs. Therefore, we recommend not to use feeder cells that have been stored in liquid nitrogen for more than 3 months.
• Inappropriate size of pieces of colonies when picking decreases the viability of iPSCs. It is important that the fragments of iPSC colonies have the appropriate size (small clumps of ∼175 μm), as big clumps (>250 μm) cannot adhere, and small ones (<100 μm) do not survive well.
• We found that the recovery rate after passaging was improved when keeping the plates undisturbed in the incubator for 48 h before refreshing the culture medium. In addition, when hESC medium of each well is changed, not all the medium is aspirated but ∼0.5 mL of medium is left before adding 2 mL of fresh hESC medium.

### Maintenance and stocking of iPSC cultures

**Figure 3:**
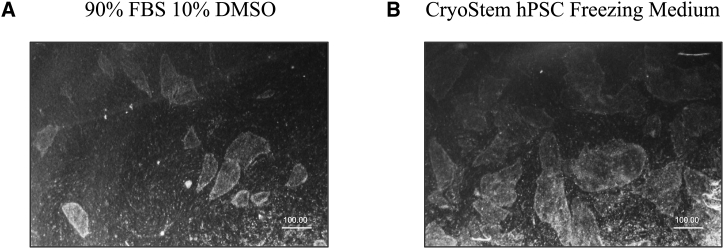
Comparison of iPSC freezing media Images from stereomicroscope of (A) colonies growing onto feeder cells, which appeared 15 days after thawing when using freezing medium consisting of 90% FBS and 10% DMSO. (B) Colonies growing onto feeder cells, which appeared 4 to 5 days after thawing.

Both the freezing protocol employing CRYOSTEM hPSC freezing medium and the one employing freezing medium consisting of 90% FBS and 10% DMSO showed good recovery rates after thawing. To note, when iPSCs were frozen using the freezing medium (90% FBS and 10% DMSO) the recovery rate was slightly lower, and if colonies finally appeared in culture, they appeared as late as 15 days after thawing (Figure 3A). On the contrary, when using the CRYOSTEM, compact colonies begun to grow 4–5 days after thawing (Figure 3B). Considering this, the freezing protocol was established using the CRYOSTEM and following the instructions described in the methodology section, but both protocols are suitable for cryopreserving iPSCs, as previously reported.^31^

#### Troubleshooting

Problem:

• From very low to non-recovery of the iPSCs after thawing.

Possible reasons/solution:

• Make sure that only healthy colonies are cryopreserved and avoid picking differentiated areas. Differentiated areas should not exceed 20% of the well if the culture is of high quality.**^32^**
• To increase the number of cryopreserved colonies per vial may be needed. In our experience, cryopreserving <10 iPSC colonies leads to almost non-recovery of the cells after thawing. Ideally, one complete well of healthy colonies is recommended to ensure recovery after thawing. Leftovers from wells already picked cannot be considered healthy colonies even when the morphology resembles them. If still unsuccessful, we recommend testing different densities of freezing/thawing (e.g., one well of a six-well plate, two wells of a six-well plate, or three wells of a six-well plate).
• Ensure that the fragments of the iPSC colonies have the appropriate size (small clumps of ∼175 μm) since dissociation to single cells decreases the viability.
• Perform both freezing and thawing processes quickly but gently.
• We have found it to be crucial to use freezing containers that enable a slow rate-controlled cooling protocol, such as the CoolCell.

### iPSC culture on feeder-free conditions

#### Tested protocols

A number of combinations were tested as summarized in Table 1. Matrigel-coated plates together with cHFF medium were used for the first adaptation attempt. After the first passage, performed mechanically with a stripper micropipette, the iPSC fragments adhered to the plate, appearing under the microscope as small clusters of compact cells that grew maintaining the rounded colony morphology. Nonetheless, when performing the next passaging—both mechanically, using 0.05% trypsin-EDTA or Versene—the colony fragments did not adhere to the plate and remained as floating fragments. Addition of 1 μL/mL (10 μM) of ROCK inhibitor Y-27632 to the culture medium improved survival and adherence of the iPSC colonies fragments when passaged as previously reported,^33^ except when using trypsin-EDTA. However, from the fourth passage onward, the colony fragments remained suspended in the culture medium and did not adhere to the plates. Therefore, it was not possible to either grow or expand iPSCs with this system.

**Figure 4:**
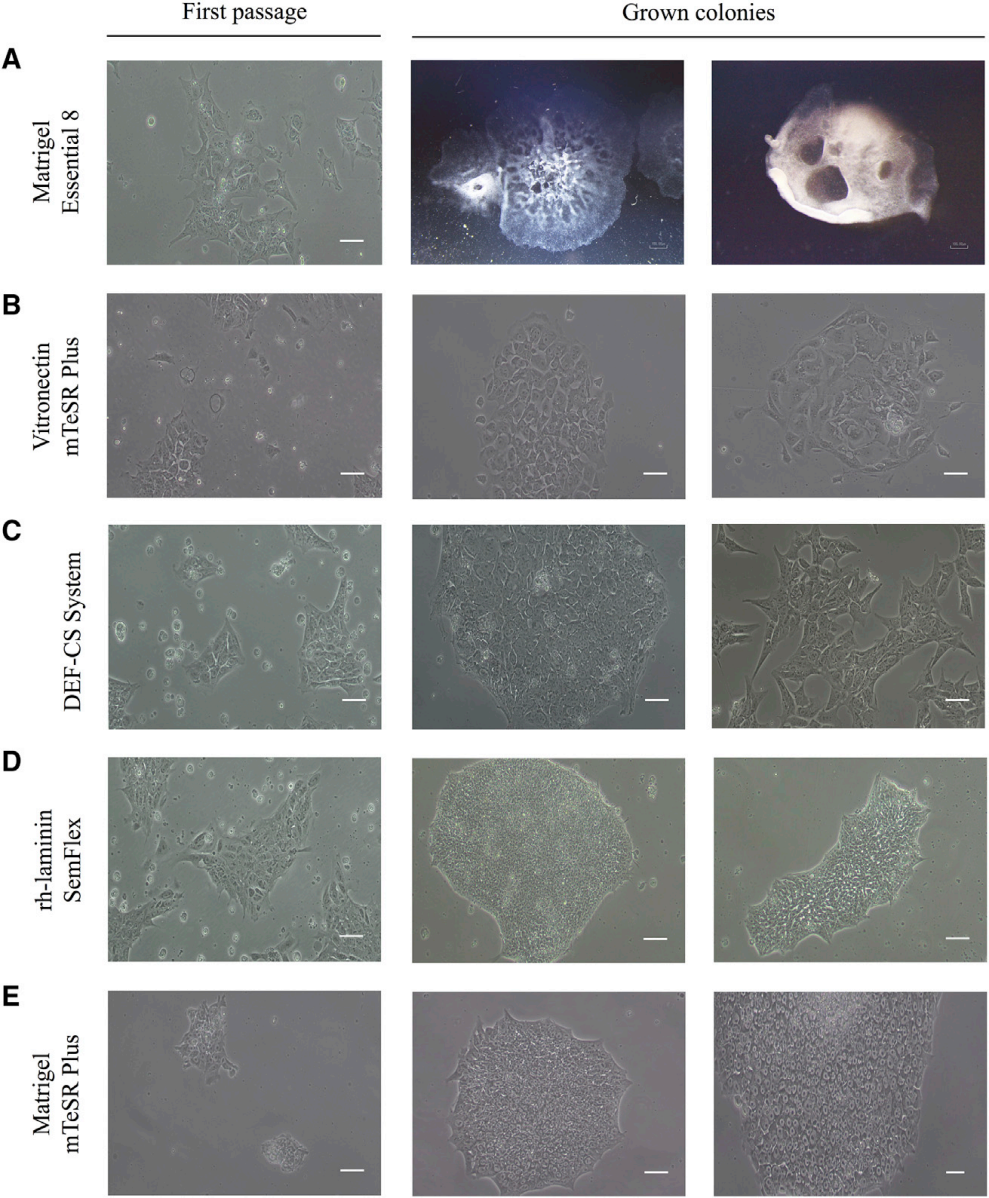
Adaptation to feeder-free culture conditions Representative images of the results obtained when testing different combinations of matrices and culture media to adapt iPSCs to feeder-free culture conditions. (A) Matrigel in combination with Essential 8 medium. (B) Vitronectin in combination with mTeSR Plus medium. (C) DEF-CS system. (D) rh-Laminin-521 in combination with StemFlex medium. (E) Matrigel in combination with mTeSR Plus. Second and third image of row (A) were taken with SMZ-745T stereomicroscope coupled to a real-time DS-FI2 photomicrograph camera. The rest of the images were taken employing an Olympus BX51 M microscope coupled to an Olympus DP70 digital camera. Scale bars, 100 μm.

The commercial culture medium Essential 8 was next used in combination with Matrigel-coated dishes. On the first passage, culture medium was supplemented with 10 μL/mL of RevitaCell 100X, as recommended by the manufacturer (Figure 4A). In this case, adhered iPSC colony fragments were observed. When iPSCs proliferated in culture and colonies increased in size, differentiated areas appeared, especially in the central part of the colonies, which occasionally formed spontaneous embryoid bodies (EBs).

Two more adaptation tests were performed using Essential 8 medium, both supplemented and non-supplemented with 10 μL/mL of RevitaCell 100X, in combination with two different matrices: vitronectin and Geltrex. In these two systems, colony fragments did not adhere to the plates after the first manual passage. The same results were observed when the Geltrex matrix was combined with the StemFlex culture medium. When vitronectin was used in combination with mTeSR Plus (Figure 4B), the cells adhered to the culture dishes, but debris and differentiated cells were observed. Grown colonies were not compact and disappeared with time.

In the DEF-CS culture system, iPSCs cells grew as rounded colonies after the first manual passage (Figure 4C) and it was possible to perform subcultures both mechanically and enzymatically. However, colonies of some clones lost the ESC-like morphology characterized^30^ by the high nucleus-to-cytoplasm ratio, defined borders, and prominent nucleoli, rather tending to spread out.

When the iPSC clones were adapted to the culture system consisting of a rh-laminin-521 matrix and the StemFlex culture medium (Figure 4D), high cell death and abundant colony fragments suspended in the culture medium were observed after the first manual passage. Once the medium was refreshed, the small groups of cells that had attached to the plates began to proliferate as colonies with defined borders and compact morphology. From 5 to 6 days after the first passage, a second manual subculture was performed. The third subculture was carried out 5–6 days later, using the Versene enzyme, and iPSCs proliferated with the typical undifferentiated ESC-like colony morphology. In addition to these recommendations, the two first passages in feeder-free conditions were performed manually with the stripper micropipette. Once the cells had been enzymatically subcultured for 3 to 4 times, cells were seeded in 1:20 proportion to ensure that iPSC colonies grew undifferentiated until 90% confluency was reached. StemFlex culture medium was supplemented with 10 μL/mL of RevitaCell 100X only when cells were passaged. Given the good results obtained, we initially consider this protocol to be implemented for culturing and expanding the iPSCs in feeder-free conditions. However, colonies of some clones showed a peculiar “half-moon” shape morphology (Figure 4D, third image) and formed sporadically spontaneous EBs.

Finally, we tested a similar protocol but with a different combination of culture media (mTeSR Plus) and matrix (Matrigel): (1) the two first passages in feeder-free conditions were performed manually with the stripper micropipette; (2) from the second passage onward, iPSCs were subcultured by using gentle cell dissociation reagent; (3) culture medium was supplemented with 10 μL/mL of RevitaCell 100X only when cells were passaged. Using this method, colonies with defined borders and compact morphology were obtained, and low cell debris and differentiation are observed (Figure 4E). Therefore, it was the finally established method for culturing and expanding the iPSCs in feeder-free conditions.

#### Troubleshooting

Problem:

• iPSC fragments do not attach to the plates after passaging.
• The main part of the iPSC colonies growing on feeder-free conditions start to differentiate.

Possible reasons/solution:

• Appropriate size of the iPSC colony fragments is essential. If the clumps are too big (>200 μm), the cells will not be attached to the plate and they will tend to form spontaneous EBs. On the contrary, too small fragments (<100 μm) or single cells^34^ are more prone to differentiation.
• We have observed that performing the first two to three passages in a manual way improves cell survival and adaptation efficiency. In addition, supplementation of the culture medium with RevitaCell 100X or ROCK inhibitor Y-27632 may also improve survival and adherence of the iPSC colonies fragments when passaged.^33^ To note, a certain number of differentiated cells in the culture is normal, especially during the first passages. These cells should disappear with medium changes.
• Revise the concentration of the coatings employed since empirical determination may be required as suggested by the manufacturer's instructions.
• Difficult-to-adapt iPSC lines may be affected by the change of the culture medium when transferred to a feeder-free system. Performing a gradual transition combining the culture medium used in the feeder-based system with the culture medium of the feeder-free system (1:1 proportion) could help to diminish this risk.
